# Explaining risks and benefits of loco-regional treatments to patients

**DOI:** 10.1016/j.breast.2023.08.006

**Published:** 2023-08-22

**Authors:** Ana-Alicia Beltran-Bless, Stephanie Kacerovsky-Strobl, Michael Gnant

**Affiliations:** a Division of Medical Oncology and Department of Medicine, University of Ottawa, Ottawa, Ontario, Canada; b Breast Health Center, St. Francis Hospital, Vienna, Austria; c Comprehensive Cancer Center Vienna, Medical University of Vienna, Vienna, Austria; d Austrian Breast and Colorectal Cancer Study Group, Vienna, Austria

**Keywords:** Locoregional, Breast cancer, Breast conserving therapy, Mastectomy, Radiation

## Abstract

Treatment for early-stage breast cancer is complex, requiring multidisciplinary care with a multitude of treatment options available for each patient. Coupled with the rising importance of shared decision-making, patient-physician conversations are progressively more complicated. These conversations require frank disclosure of risks and benefits of the different treatment modalities in a way that is individualized for each patient and simple to understand. In most patients, breast conserving therapy with radiation should be presented as the gold-standard local treatment given similar long-term and improved quality of life outcomes. De-escalation is currently at the forefront of research in loco-regional treatments, and further investigations are required to best determine the optimal patient populations for reduced sentinel lymph node sampling, omission of sentinel lymph node biopsy altogether and omission of radiation treatment. For future trials, better endpoints need to be established considering patient-centered outcomes as well as recurrence.

## Highlights D-23-422

• Locoregional treatment should be based on shared decision-making
• Physicians need to fully disclose all risks and benefits of local treatment
• Outcomes of breast conserving surgery are similar/improved compared to mastectomy
• Breast conservation ist he standard of care!

## 1 Introduction

The concept of shared decision-making is now at the forefront of oncologic management. The past few decades have shown a shift from a more paternalistic healthcare model to patient-centered care allowing patients more autonomy and active participation in their own care. The unlimited wealth of information available at patient's fingertips combined with the current complexity of cancer care requiring a high degree of individualization has led to more challenging discussions for both patients and physicians. Besides ethics, the pragmatic hope behind shared-decision-making is to improve equity in healthcare service and delivery, enhance the process of care, optimize psychological recovery, increase patients' treatment adherence, and improve treatment satisfaction [1]. Shared decision-making is believed to be achieved when two objectives are met: patients are fully informed of treatment options, risks and benefits and patients' values and preferences are incorporated into decisions [2]. Patient preferences are defined as a patient's perspectives, beliefs, expectations, and goals for their health and life [3]. Little is known on how patients construct and express preferences, what structure underlies them and how their importance is determined. Intuitive judgement is felt to dominate the construction of preferences after a cancer diagnosis, with availability bias and risk aversion strongly influencing patient perspectives [2]. The impact of key decisional supports such as family and clinicians on patient decision-making is not well elucidated [4].

As physicians, there is an ethical obligation to disclose all information required by patients in the process of shared decision-making, however this information needs to be tailored to the needs of the individual patient. It is known that despite patients having a high desire for information, some patients feel underinformed or overwhelmed by the amount of information that is shared [5]. Patients need to be aware of true complication rates and morbidity related to their oncologic treatments, as well as expectations on how the process will unfold.

Breast cancer is the most prevalent cancer worldwide, with 2.3 million women diagnosed and 685 000 deaths in 2020 [6]. In addition to improvements in breast cancer diagnosis in past decades, the local management and efficacy of systemic therapies has significantly changed. A one-size fits all approach no longer works for patients, and many options may exist for both local control and systemic therapy in each patient. Local therapy for breast cancer consists of surgical resection with sampling or removal of axillary lymph nodes, with potential postoperative radiation. Goals of local therapy in early-stage breast cancer remain to achieve local control, prevent recurrence, and improve overall survival. Given excellent breast cancer outcomes in most patients, there is continued importance on reduction of morbidity, improved aesthetic outcomes, and improved quality of life. In this review, we will focus on the current evidence behind loco-regional breast cancer treatments, and review risks and benefits to help guide patient discussions.

## 2 Breast conserving therapy vs mastectomy

Local surgical resection options include breast conserving therapy (BCT) and mastectomy. It is known that breast conserving therapy followed by postoperative radiotherapy (RT) has shown no difference in disease-free (DFS) and overall survival (OS) compared to mastectomy in many randomized clinical trials [7,8]. More recent retrospective studies on large patient populations have shown that BCT and RT might lead to better breast cancer specific survival (BCSS) and OS than mastectomy alone [9,10]. Local recurrence rates have historically been quoted as higher with BCT + RT, however with better systemic and radiation treatments they are now shown to be as low as after mastectomy [[11], [12], [13]]. Improvements in adjuvant systemic therapy have also led to a reduction in locoregional recurrences by half between 1990 and 2011 [14]. Given clinical equipoise between BCT and mastectomy, decision-making is now increasingly based on well informed patient discussion and patient preferences. In order to conduct a successful BCT, the tumor must be safely removable with an acceptable cosmetic result followed by safe delivery of radiotherapy. Contra-indications to BCT can be summarized in three indications: 1) the area of disease to be removed is too large (e.g. multicentric disease, diffuse malignant calcifications on imaging), 2) ineligibility for radiation (e.g. prior RT, pregnancy, connective tissue disease) and 3) persistent positive margins [15]. Although more recently, multicentric disease is no longer seen as a clear contraindication to BCT with results from the ACOSOG Z11102 trial [16].

Radical mastectomies have become more conservative through various techniques including skin preservation (skin sparing mastectomy) and nipple-sparing (NSM). It is known that quality of life is superior in patients with a mastectomy whose reconstruction is performed with autologous flaps and when nipple-sparing is used [17]. Medical indications for mastectomy are present only in a minority of patients, and despite improved breast cancer outcomes over time including a decrease in contralateral tumors [18], bilateral mastectomy rates for unilateral cancers have been documented to be on the rise in the US and Canada [12,19]. It is unusual that while oncology strives for improved personalized and de-escalated breast cancer treatments, patients desire for mastectomy is on the rise. Evidence points to this rise in mastectomies being patient-driven and stemming from concerns regarding fear of disease recurrence [12]. This is potentially related to the shift from paternalistic care, and more impact of patient preferences. Analysis shows the importance of physician discussion and recommendations in this decision, as among patients who underwent contralateral prophylactic mastectomy, 37.3% believed it would improve their survival, and few patients proceeded with the procedure when their surgeons recommended against it (3.7%) [20]. This evidence underscores the importance of efficient communication, patient education and evidence-based decision-making with the patient prior to surgery.

Fear is an important variable that plays a large part in patient perceptions and decision-making and is not easily cured by any specific surgical technique. Physicians have an ethical obligation to educate patients, ensuring that the optimal treatment modality is well explained to patients, and that they properly understand benefits and harms. Patients need to be clearly informed that mastectomy is not associated with lower disease recurrence or improved survival. Contralateral mastectomy also does not improve overall survival but does lead to increased surgical complications [21]. Patient's age, lifetime risk and increased risk of surgical complications should come into play in any discussion on prophylactic mastectomy [12]. While surgical reconstruction should be offered to all patients who proceed with a mastectomy, a realistic discussion regarding outcomes needs to be had such as cosmesis, healing time, risk of repeat surgery and a close to 30% complication risk [22,23]. Patients tend to be informed regarding advantages more than disadvantages of treatments. A 2016 study showed that patient knowledge regarding risk of complications of reconstructive surgery was low with only 15% correctly estimating the risk, with the remaining patients underestimating the risk [24]. Patients also must be aware that immediate reconstruction with postoperative complications are associated with oncologic treatment delays [25].

An exception exists in patients with deleterious BRCA mutations. Prophylactic bilateral mastectomy should be discussed in these patients as a potential way to reduce the high life-time risk for developing breast cancer. In patients with BRCA mutations who are already diagnosed with breast cancer, BCT is generally safe with no impact on survival. In patients with BRCA mutations, contralateral prophylactic mastectomy reduces the risk of contralateral breast cancer but does not improve overall survival [13].

The usefulness of neoadjuvant systemic therapy to downstage tumors and axillary lymph nodes eventually facilitating BCT has been documented. This surgical de-escalation - increasing patient's eligibility for BCT - yields equivalent long-term outcomes compared to primary BCT [26,27]. The higher local recurrence rate in patients who were initially planned for a mastectomy and underwent BCT in the EBCTCG meta-analysis was interpreted as a result of outdated imaging and margin assessment, as well as the potential omission of surgery in patients with a complete response [26]. BCT conversion rates after neoadjuvant treatment are high in literature [28,29]. Despite these responses, we are still limited by uptake of these practices and false size predictions from pre-operative imaging. Results from the BrighTNess trial demonstrated BCT conversion in half of patients with stage II and III TNBC, however one third of BCT eligible women still underwent mastectomy [30]. In patients deemed ineligible for BCT after neoadjuvant treatment, pathologic complete response was documented in one third. A recent abstract presented at ASCO showed that 52.3% of patients who underwent mastectomy after neoadjuvant chemotherapy were actually BCT eligible [29]. Thus, indicating a requirement for more accurate measures of response to therapy.

For patients facing the challenging decision between breast conservation surgery and mastectomy, clear discussions of risks and benefits must be had. Post-surgical complications are similar, although higher post mastectomy [31], and include seromas/hematomas, wound complications and infection, pain, and problems with shoulder mobility. Important differences between the two surgeries, as well as oncoplastic techniques center on cosmetic outcome, psychological adjustment, and quality of life. It is generally accepted that patients undergoing BCT or breast reconstruction have improved quality of life outcomes compared to those undergoing mastectomy [17]. Women with BCT have reported better physical and role functioning, more sexual activity and more satisfaction with their body image compared to those who underwent mastectomy [32]. Thus, we are increasingly turning to the concept of oncoplastic surgery whereby plastic surgery techniques can be used after BCT or mastectomy to improve the aesthetic result of oncologic surgery. Oncoplastic surgery can also help patients who are not initial candidates undergo BCT, such as tumors larger than 5 cm with localized skin infiltration and multifocal tumors. These techniques can avoid unnecessary mastectomies and reconstruction.

In the decision regarding surgical management of breast cancer, patient education is paramount. Numerous studies have shown that patients who take an active role in decision-making are significantly more satisfied with their decision [33]. Decision aids have been advocated to promote shared decision-making by standardizing and simplifying communication between patients and physicians. Decision aids on the choice of breast cancer surgery have been studied in Canada, the United States and the Netherlands [34]. Interviews of physicians in the UK in 2010 showed noted benefits of a potential decision aid, however expressed concern about information, overload and content [1]. Many physicians voiced desire to restrict information related to survival, and prognosis and to supervise patient's access to the decision aid to minimize distress and anxiety. More research into the use of decision aids to aid physician discussion regarding surgical procedures, prognosis and outcome is required, as well as better ways to promote patient education.

## 3 Surgical management of the axilla

Surgical management of axillary lymph nodes is assessed separately from surgical therapy of the breast. It acts as both a diagnostic and prognostic procedure, by determining the anatomic extent of spread, and a therapeutic purpose, by removing all evident cancer cells. Decisions regarding surgical procedures is based on whether axillary lymph node involvement is clinically evident at diagnosis, and whether neoadjuvant treatment is received. It is important to have discussions with patients regarding morbidity associated with axillary surgery and the various surgical options.

For clinically node negative patients, sentinel lymph node biopsy (SLNB) is now considered standard of care. SLNB requires injection of a tracer into the breast, whereby lymphatic drainage is used to locate and remove axillary lymph nodes, identifying the first few lymph from which the tumor drains for pathologic examination. Studies have shown that SLNB negativity in the clinically node negative population leads to equivalent regional control, DFS and OS as axillary lymph node dissection (ALND) [35]. Compared to the previous widespread use of ALND, SLNB is associated with less morbidity, such as reduction in lymphedema, hemorrhage, infection, seroma, neuropathy with chronic pain, and shoulder movement impairment with associated improvement in quality of life [36,37]. The reduction of morbidity, with equivalent outcomes led to the widespread acceptance of SLNB as axillary staging in the clinically node negative population worldwide. For patients with T1-T2 tumors with positive SLNB results, the ACOSOG Z0011 and IBCSG 23-01 trials were conducted. These trials demonstrated that in patients with one to two positive sentinel lymph nodes who underwent whole breast irradiation, completion ALND can be safely avoided [38,39]. Of note, later analysis of the radiation fields received in Z0011 found that a non-trivial amount (18.9%) of patients received directed nodal RT via a third field [40].

ASCO guidelines were amended in 2014 to recommend against ALND for patients with fewer than 3 sentinel lymph nodes [41]. Completion ALND is still recommended for patients who do not fit the Z0011 criteria, including patients with inflammatory breast cancer, three or more pathologically involved sentinel lymph nodes and for patients with extranodal extension. Some evidence exists that the degree of extranodal tumor extension is important, with recommendation that only patients with >2 mm should be considered for ALND however this is not based on randomized evidence [42,43].

Patients with clinically positive, biopsy confirmed axillary disease who do not undergo neoadjuvant treatment, should have ALND. For clinically node positive patients who have received neoadjuvant therapy, choice of surgical management depends on the extent of nodal involvement prior to treatment and degree of response to treatment and includes SLNB, ALND and/or axillary radiation. There is no consensus on axillary management for clinically node positive patients after neoadjuvant therapy and this continues to be an active area of investigation. Patients with clinically palpable nodes should have these biopsied and clipped prior to neoadjuvant treatment. Patients with cN1 with no evidence of residual nodal disease should be considered for a SLNB and removal of clipped lymph node with retrospective studies showing few axillary recurrences in this population [44]. Despite this evidence, a survey in 2022 saw that the most common post-neoadjuvant axillary surgery in cN1 patients converting to ycN0 was targeted axillary dissection (54%) followed by SLNB alone (21%) [45]. Patients with cN2-N3 on initial presentation and patients with residual disease after neoadjuvant chemotherapy require ALND.

The monarchE trial evaluated the benefit of the addition of abemaciclib, a CDK4/6 inhibitor, to endocrine therapy for two years on invasive disease-free survival in patients with high-risk hormone receptor positive, HER2 negative node positive breast cancer [46]. High-risk was defined as either greater than or equal to four positive axillary nodes or one to three positive axillary nodes with at least tumor size greater than or equal to 5 cm, histologic grade 3 disease or a ki-67 greater than 20%. This study revealed a significant improvement in invasive disease-free survival with the addition of abemaciclib. It is unclear if patients with node positive disease require completion ALND to determine total nodal burden given the possible impact on systemic therapy recommendations [47]. Thus, the avoidance of ALND as is the current standard of care is possibly leading to suboptimal adjuvant therapy.

There is now further interest in de-escalation with the avoidance of any axillary surgery in specific populations by use of medical imaging. The results of the SOUND trial recently presented at the 18th St.Gallen International Breast Cancer Conference in March 2023 looked even further at no axillary surgery [48]. Patients with negative axillary ultrasound and small (<2 cm) tumors who were planned to undergo BCT + RT were randomized to SLNB vs no axillary surgery. Outcomes at 5 years showed non-inferior disease-free survival. The details regarding radiation treatment planning, however, were not presented and leaves some questions regarding the fields of radiation and whether they were similar in the two arms in this open label trial. One factor of importance in current surgical de-escalation trials is that radiation therapy is left to the discretion of the physicians, and it is possible that radiation oncologist might be disposed to treat patients in the non-surgical arm with higher tangents to include axillary level I/II.

While de-escalation to minimize morbidity is important, it is also vital to ensure patients are not being harmed by the newest trends and by physician pressure to be progressive. By not sampling sentinel lymph nodes, or by sampling too few sentinel lymph nodes, we are unable to properly stage patients. There is clear evidence of surgeon variability in the number of sentinel lymph nodes removed in the context of a lack of standardization of this procedure [49]. This incomplete staging impacts adjuvant therapy decisions, namely the decision regarding chemotherapy and CDK4/6 inhibitors. The recurrence risk and associated systemic therapy in patients who have one out of one positive sentinel lymph node, is very different than a patient who has one out of three or three out of three. Thus, with new data on lymph node status guiding adjuvant treatment, surgeons have to be cautious and reflect on how their surgery could impact future treatment options.

## 4 Radiation therapy

As already discussed, whole-breast RT (WBRT) is standard of care post BCT with equivalent local control and possibly better survival than mastectomy alone [[7], [8], [9], [10]]. A new standard of care has emerged with a moderately hypofractionated schedule administering similar total dose of radiation, over a shorter period of typically three to five weeks in patients not requiring nodal irradiation. This was found to have equivalent tumor control and fewer toxicities in comparison to conventionally dosed WBRT which is typically administered over five to seven weeks [50]. Ultrashort WBRT presents another alternative in a low-risk population and is administered over five days. This is based on the FAST trial which compared ultrashort WBRT to conventional WBRT in node negative patients older than 50 who underwent BCT with a tumor size <3 cm and showed no difference in moderate or marked tissue effects in the ultrashort 28.5 Gy arm [51]. The FAST-Forward trial looked at hypofractionated WBRT vs ultrashort WBRT with lower radiation doses of 26 and 27 Gy and found a nonsignificant reduction in local recurrence, with the ultrashort schedule causing milder early skin reaction and similar late adverse effects [52]. Thus, in older early-stage node negative patients post BCT, 5 fractions of RT constitute a reasonable option that is as effective for reduction of local recurrence risk and equally if not more effective for cosmesis.

There is a growing body of evidence that post-BCT RT could be omitted in the future in well selected patients to avoid unnecessary acute and chronic toxicities. The recent PRIME II trial showed higher local recurrence, but similar overall survival in patients older than 65 with <3 cm node negative, hormone receptor positive tumors who underwent BCT without RT and received adjuvant endocrine therapy [53]. This presents itself as an option to be discussed with older patients with early-stage hormone positive breast cancers. The search to identify the ideal subset of patients at low risk in whom post-BCT RT can be omitted is still ongoing.

Accelerated partial breast irradiation (APBI) is another alternative to hypofractionation to reduce the required fractions and doses to nearby organs with improvement in cosmetic results. The optimal patients for APBI are still unclear [54]. Women with low-risk breast cancer will have several options of radiation in the future including hypofractionated RT, ultrashort RT, APBI or even omission of RT entirely. This will require proper patient selection, as well as clear discussions with patients of the multitude of options, evidence behind each, and potential toxicities.

Post mastectomy radiation (PMRT) can be considered in high-risk patients based on the presence of certain risk factors with a reduction in locoregional failure and breast cancer mortality. These include > T3 cancers, positive nodes, positive margins, or extracapsular extension. PMRT leads to reduction in loco-regional recurrence and improvement in overall survival in this population [54]. There is no current prospective data guiding management of PMRT in patients who previously received neoadjuvant chemotherapy and consideration should exist in patients with residual disease and cT3-4 and cN2-3 disease [55].

Regional nodal radiation (RNI) for clinically node positive patients post mastectomy is standard, with evidence of reduction in locoregional, distant recurrence and improvement in overall survival [[56], [57], [58]]. For patients with node-positive or high-risk node-negative breast cancer who undergo BCT, RNI has been associated with improves outcomes [59,60]. Data has been growing supporting the role of RNI in lieu of ALND. The AMAROS trial included patients with early-stage breast cancer, clinically node negative who were randomized in the case of a positive sentinel lymph node biopsy to ALND or RNI. Outcomes were the same in terms of axillary recurrence, DFS and OS, but there was significantly less morbidity with RNI [61]. Evidence of RNI in patients who convert to pathologic node negative is not clear. For patients who are having omission of surgical management of the axilla, for example in the SOUND trial, radiation treatment was left to the discretion of the physician, thus further information on radiation planning in this context is required.

Discussions on risks and benefits of radiation are required for breast cancer patients. For patients who express fear of receiving radiation after BCT, a pre-operative consultation with radiation oncology will be beneficial. Older patients with early-stage node-negative breast cancer should be presented the options of ultrashort and omission of radiation with the associated evidence. Radiation treatments have evolved in the last decade, with more precise delivery of radiation minimizing side effects and dosage to internal organs. Despite this, short and long-term effects are still present and require proper discussion with patients [62]. Short-term side effects include fatigue, radiation dermatitis and increased risk of infection and pain. Long-term side effects can include cosmetic changes, lymphedema, breast fibrosis and necrosis, rib fracture and neuropathy. More rare but severe toxicities come from the damage to internal organs such as pneumonitis, cardiovascular disease, compounded by the receipt of cardiotoxic systemic therapies, and the risk of second primary cancers. Given the evolution of radiation therapy in the last decade, there is likely less late toxicity with modern radiotherapy techniques. The risk of cosmesis should always be discussed, specifically in patients who are undergoing breast reconstruction, as it is accepted that radiation after immediate breast reconstruction increases the risk of additional reconstructive surgery [63].

## 5 Discussion

Physicians need to acknowledge that a large part of patient perceptions are influenced by fear and that patients who report concerns about recurrence are more likely to proceed with a mastectomy [63]. This is likely largely cemented on previous belief that mastectomy leads to improved outcomes as a survey found that when patients underwent prophylactic contralateral mastectomy, 37.3% felt that it would lead to improved survival and 96.3% endorsed peace of mind as their motivation [64]. Risk aversion and anticipated regret also play a large part in patients desire for maximal interventions [2]. Physicians instead need to review current evidence of similar local and overall survival but improved quality of life associated outcomes with BCT and RT. For patients who chose to undergo mastectomy, frank decisions are required regarding complications of surgery and reconstruction. It should be the physician's responsibility to assess the reason for the patient's decision, if it is fear, disinformation, prior consultation of other physicians who are not familiar with the state-of-the-art breast cancer treatment strategies. All of this leads to an intense and time-consuming wrap-up, but facing the alternative, the mastectomy, which can't be reversed after performed, it is worth the time. Surgical management of the axilla continues to be controversial given the desire to reduce surgical morbidity being offset by a desire for accurate staging information and adjuvant therapy. In older patients with early-stage node negative breast cancer, ultrafractionation or hypofractionation after BCT and its potential benefits need to be reviewed. Omission of RT should be decided on a case-by-case basis in older hormone receptor positive early-stage patients.

Innovative study endpoints for optimal management of breast cancer patients in the curative setting need to be established. It is well accepted that the goal of both locoregional and systemic treatments are to cure patients with early breast cancer but - given improved outcomes in this patient population - other patient-centered outcomes need to be assessed. Uncertainty arises regarding how best to measure these patient outcomes. Many researchers have attempted to create standardized approaches for cosmetic evaluation after BCT. This has been done subjectively by the surgeon, patient themselves, an unrelated third party, with photography and defined measurements, or scoring based on semi-objective subscales [65]. The optimal assessment technique for aesthetic outcomes has still not been established. Patient-reported-outcome measures (PROs), defined as a patient's self-report on the status of their health condition, are increasingly being used in medical oncology. They allow healthcare providers to understand their patient's view of their healthcare interventions. Pre-treatment PROs have been shown to predict benefits and harms of breast cancer therapies. PROs are being recognized in their ability to help individualize treatment and facilitate informed decision-making [66,67]. They have been used specifically to measure breast surgery outcomes and concentrate on postoperative patient satisfaction with their breasts [68,69]. The future might see PROs being incorporated into performance measures, and reimbursement [70]. However, PROs are limited in their subjectiveness and low reproducibility. PROs can also be incorporated into composite endpoints. Composite endpoints allow for multiple domains to be added into a single output. Attempts have already been made to pilot a composite endpoint of BCT including patient self-reported cosmetic outcome [71]. Allowing each patient to weigh the importance of individual domains in the composite endpoint through PROs pre-operatively is likely only one future avenue of shared decision-making. While substantial research already exists on the use of artificial intelligence (AI) in breast cancer imaging and pathology, its use in local breast cancer management is not as developed. In terms of cosmetic outcomes, computer programs have been created to objectively measure postsurgical aesthetics. Machine-learning is felt to be the next step to integrating this information into a global aesthetic evaluation [72]. AI is also under investigation to help intra-operative localization of cancer and in breast reconstruction [73]. AI will continue to be present in healthcare, and the goal should be to augment the quality of care that is delivered.

One future problem that will need to be addressed is how best to address information patients can access on the internet into an optimized physician-patient interaction. Compiling this information and providing patients with high-quality resources prior to their clinical visit could be a way to mitigate their access to misinformation. These guides could include websites which contain information on epidemiology, prognosis, treatment options and toxicity, as well as support groups.

Both the systemic and local management of breast cancer has become increasingly complex with the advent of new techniques and new treatment modalities. Patients desire involvement with their physician in decision-making and do not want their physician making these decisions alone [5]. More than ever before, treatment decisions should be based on shared decision-making between the patient and physician, as well as multidisciplinary discussion. Information related to benefits and current evidence, need to be shared in a way that is understandable and tailored to each patient. As breast cancer outcomes continue to improve, further research needs to be put into improving quality of life for patients through de-escalated local treatments to improve cosmesis and minimize long term side effects. However, de-escalation cannot come at the cost of suboptimal systemic treatment. As we shift to personalized oncologic treatments, the decision regarding escalation and de-escalation of treatment requires accurately identifying appropriate subsets of patient. In the future, the use of clinical risk factors, nomograms, genomics, and biomarkers will guide the appropriate selection of patients for de-escalated treatments. A fine balance must be reached in the de-escalation of treatments between the treatment morbidity and benefits.
